# Metabolomics Reveals Resistance-Related Secondary Metabolism in Sweet Cherry Infected by *Alternaria alternata*

**DOI:** 10.3390/biom15121730

**Published:** 2025-12-12

**Authors:** Huaning Yuan, Hanfeng Gao, Shupeng Duan, Xiaoyu Zhou, Xiuru Yang, Bo Sun, Hongwei Ren, Zhenzhen Zheng, Qingyun Guo

**Affiliations:** 1 Qinghai University, Xining 810016, China; 19809797623@163.com (H.Y.); gaohanfenglz@163.com (H.G.); 13772381896@163.com (S.D.); hzlzdal@163.com (X.Z.); wyb97852024@163.com (X.Y.); 2 Academy of Agriculture and Forestry Sciences, Qinghai University, Xining 810016, China; 3 Key Laboratory of Agricultural Integrated Pest Management in Qinghai Province, Academy of Agriculture and Forestry Science, Qinghai University, Xining 810016, China; 4 College of Horticulture, Sichuan Agricultural University, Chengdu 611130, China; bsun@sicau.edu.cn; 5 Academy of Agricultural and Forestry Sciences in Luoyang, Luoyang 471023, China; renhongweijjl@sohu.com; 6 Key Laboratory of Qinghai-Tibet Plateau Biotechnology, Ministry of Education, Qinghai University, Xining 810016, China

**Keywords:** sweet cherry, brown spot disease, disease-resistant cultivar, metabolomics

## Abstract

Sweet cherry (*Prunus avium* L.) is a tree species cultivated worldwide with high economic value. During its growth, it is frequently threatened by pathogenic fungi, leading to reduced yield and deteriorated quality. However, in sweet cherry, the disease-resistant cultivars against brown spot disease (BSD) caused by *Alternaria alternata*, as well as the associated disease-resistant metabolic pathways and metabolites, remain limited. In this study, we investigated the disease-resistant germplasm and associated metabolic pathways of sweet cherries using field disease resistance screening, physiological analyses, and metabolomics. The results showed that sweet cherry cultivar Q8 exhibited relatively strong resistance to BSD, while cultivar Q9 demonstrated the weakest resistance. Physiological experiments revealed that the changes in relative electrical conductivity (REC), malondialdehyde (MDA) content, and relative water content (RWC) of the disease-resistant cultivar Q8 were smaller than those of Q9 within 9 days under *A. alternata* infection. At 9 days post-infection (dpi), the relative electrical conductivity (REC) of Q9 (57.78%) was significantly (*p* < 0.05) higher than that of Q8 (49.01%), whereas the relative water content (RWC) of Q8 (78.29%) was significantly (*p* < 0.05) greater than that of Q9 (67.88%). For malondialdehyde (MDA) levels, Q8 reached its peak value (27.81 nmol/g) at 3 dpi, while Q9 attained its maximum (27.80 nmol/g) at 9 dpi. At 3 dpi and 9 dpi, Q8 consistently maintained a significantly (*p* < 0.05) higher RWC than Q9. Linolenic acid metabolism and unsaturated fatty acids were found to be involved in the disease resistance process, and Pinellic acid might be a bioactive metabolite conferring disease resistance. The disease-resistant metabolic pathways and bioactive metabolites identified in this study may be conserved in plants beyond sweet cherry, providing a theoretical basis for disease-resistant breeding of sweet cherry.

## 1. Introduction

Sweet cherry is a fruit tree of the Rosaceae family and has high commercial value [1]. Because of its attractive blossoms, desirable flavour, and high nutritional value, sweet cherry is cultivated worldwide [2]. Metabolomics is an analytical approach that quantifies small-molecule metabolites involved in cell structure, signal transduction, and numerous physiological processes [3]. Metabolomics plays a crucial role in plant stress resistance, disease resistance, quality analysis, and biological mechanism elucidation [4,5,6].

Current research related to sweet cherry mainly focuses on the regulation of fruit size and its mechanism, peel color, cold tolerance, fruit quality, salt tolerance, fruit softening, and antioxidant capacity of fruit [7,8,9,10,11,12,13]. During the growth of sweet cherry, it is frequently threatened by pathogenic microorganisms, which leads to yield reduction, mildew, fruit wilting, and other problems. In the group’s early work, severe BSD was found in sweet cherry planting areas in Haidong City, Qinghai Province, China. With identification of the pathogenic fungus by morphology and molecular biology, the disease was confirmed to be caused by *A. alternata*. Although numerous studies have reported that sweet cherry can be infected by pathogenic microorganisms such as fungi and viruses [14]. For example, cherry downy mildew caused by *Peronospora clandestina* (Wall.) Lev. is a severe disease infecting American sweet cherry cultivars (Bing and Sweetheart), which seriously threatens their quality [15]. Soft rot caused by *Alternaria* spp. has previously led to significant yield reduction of Asian sweet cherry [16]. It has also been reported that virescence disease caused by pathogenic strains related to *Candidatus Phytoplasma ziziphi* occurs in China. Diseased sweet cherry exhibits wilting symptoms, and infected flowers fail to produce fruit, ultimately leading to plant mortality [17].

However, reports on the endogenous metabolite changes of sweet cherry under pathogenic infection stress and on the key metabolic modules involved in pathogen defense are absent. In this study, 34 commercial cultivars were evaluated under field conditions; Q8 displayed strong resistance, whereas Q9 was highly susceptible. Physiological experiments revealed that REC, MDA and RWC contents between Q8 and Q9 showed significant differences at different infection points. Specifically, the REC increased significantly in both cultivar Q8 and Q9, while Q9 showed a greater range of increase; the MDA content increased initially and then decreased in cultivar Q8, while that in Q9 exhibited a continuous rise; the RWC significantly decreaseed in both cultivars, but Q9 exhibited a larger range of decrease. Metabolomic analysis revealed that linolenic acid and unsaturated fatty acid metabolisms were involved in the disease-resistance module, with Pinellic acid potentially acting as a defensive metabolite against *A. alternata* in sweet cherry.

Therefore, this study clarified the metabolic pathways involved in sweet cherry against *A. alternata* at the metabolic level, laying a foundation for exploring disease-resistant genes in sweet cherry in the future.

## 2. Materials and Methods

### 2.1. Test Materials

Experimental trees were planted at the Cherry Base of the Dunding Mountain Forest Farm in Haidong City, Qinghai Province, China (latitude 36°16′ N, longitude 102°09′ E). The site is at an altitude of approximately 1900 m, with an average annual temperature between 6 °C and 8.6 °C, and annual precipitation ranging from 330 to 450 mm. The 8-year-old trees were under appropriate fertilizer and water management and exhibited robust growth. The experiment was conducted in mid-July, and leaf samples were collected in the early morning. Middle-canopy leaves from healthy, disease-free sweet cherry trees of uniform vigor were selected for inoculation.

### 2.2. Test Strains

The pathogen causing sweet cherry BSD was isolated and identified as *A. alternata* in 2017. The pathogen was originally isolated from diseased sweet cherry leaves collected in Haidong City, Qinghai Province. Isolation was performed using the tissue isolation method. The fungal species was identified and confirmed as *Alternaria alternata* through morphological characteristics combined with sequence analysis of the ITS, *EF-1α*, and *Alt a 1* gene. The pathogenicity of *A. alternata* was confirmed following Koch’s postulates. The purified strain is maintained at 4 °C in the Qinghai Provincial Key Laboratory of Integrated Pest Management. Sweet cherry leaves infected with BSD develop circular to sub-circular brown lesions of varying sizes. Under severe disease conditions, these lesions coalesce, expand to cover the entire leaf surface, significantly impair photosynthesis, and lead to a decline in tree vigor [18].

### 2.3. Field Disease-Resistance Evaluation

The purified pathogen was inoculated onto Potato Dextrose Agar (PDA) plates and cultured at a constant temperature of 25 °C in the dark for 7 days. Mycelial discs (5 mm in diameter) were aseptically cut from the edge of the colonies using a sterile cork borer and used as inoculum. For inoculation, we selected leaves from the middle canopy of healthy, disease-free sweet cherry trees exhibiting uniform growth. One tree was sampled per cultivar. The leaf surfaces were disinfected with 75% ethanol, rinsed with sterile distilled water, and then air-dried. For inoculation, 2–6 fungal plugs were placed on each leaf. To maintain high humidity, sterile water was sprayed every 24 h. A total of nine leaves were inoculated per cultivar. Disease progression was observed on days 5, 7, 9, and 11 dpi. During each observation, lesion diameters were measured using the cross-arm method. The pathogenicity of the isolate and the disease resistance level of the leaves were evaluated based on the lesion diameters.

### 2.4. Method for Determination of RWC

Following pinprick inoculation with fungal plugs, leaves from highly resistant and susceptible cultivars were collected at 0, 3, and 9 dpi. For each sample, approximately 1 g of tissue was excised and immediately weighed to quantify the fresh weight (FW). The samples were then vacuum-infiltrated with distilled water and soaked in the dark at room temperature for 6 h. After saturation, surface moisture was blotted dry with paper towels before weighing to obtain the turgid weight (TW). Samples were then oven-dried at 105 °C for 30 min to terminate enzyme activity, followed by drying at 80 °C for 6 h. After cooling in a desiccator, the dry weight (DW) was recorded. The experiment consisted of three biological replicates per treatment and was independently repeated three times [19]. The leaf RWC was calculated using the following formula: RWC (%) = $\left(\mathrm{TW}-\mathrm{DW}\right)/\left(\mathrm{FW}-\mathrm{DW}\right)$ × 100%.

### 2.5. Determination of REC

Following the removal of midveins, the collected sweet cherry leaves were cut into uniformly sized pieces. A 0.5 g sample of the prepared leaf segments was then weighed and transferred to a clean test tube. After removing the midveins, the leaves were cut into pieces of uniform size. A 0.5 g portion of the leaf pieces was weighed and transferred into a clean test tube. Then, 20 mL of deionized water was added, and the tube was allowed to stand at room temperature for 30 min. The electrical conductivity of the solution was measured using a conductivity meter (Shanghai Yidian Scientific Instrument Co., Ltd., Shanghai, China) and recorded as *C*_1_. Subsequently, the test tube was covered (leaving a small vent) and placed in a boiling water bath for 10 min. After cooling to room temperature, deionized water was added to restore the solution to its initial volume. The electrical conductivity was measured again and recorded as *C*_2_. A blank control tube, containing the same volume of ultrapure water as the sample tubes but no plant material, was processed simultaneously and subjected to the same procedures (including standing, boiling, and cooling). Its electrical conductivity was measured and recorded as *C*_0_. The experiment included three biological replicates per treatment and was independently repeated three times [20]. The leaf REC was calculated using the following formula: REC (%) $=\left(C_{2}-C_{0}\right)/\left(C_{1}-C_{0}\right)$ × 100%.

### 2.6. Determination of MDA Content

The MDA content was determined using the BoxBio MDA Assay Kit (Beijing Box Biotechnology Co., Ltd., Beijing, China). All reagents required for the assay were provided within the kit. The specific procedures were conducted as follows: approximately 0.1 g of leaf tissue was weighed and homogenized in an ice bath with 1 mL of the provided extraction solution. The resulting homogenate was centrifuged at 8000× *g* and 4 °C for 10 min. The supernatant was then collected and kept on ice for subsequent analysis. The UV-visible spectrophotometer (Beijing Purkinje General Instrument Co., Ltd., Beijing, China) was preheated for more than 30 min. The wavelength was adjusted to 450 nm, 532 nm, and 600 nm sequentially, and the instrument was zero-calibrated using distilled water at each wavelength. Subsequently, the supernatant was transferred into a 1 mL glass cuvette, and its absorbance was measured at 450 nm, 532 nm, and 600 nm. The MDA concentration was calculated based on the absorbance values obtained at these wavelengths. The experiment included three biological replicates per treatment and was independently repeated three times [21].

### 2.7. Metabolomics Determination

#### 2.7.1. Biological Sample Preparation and Processing

Biological samples (leaf samples from Q9 and Q8 were collected at 0, 3, and 9 dpi, with three biological replicates per treatment) were placed in a freeze dryer for vacuum freeze-drying for 63 h. The dried samples were ground into powder using a grinder (RETSCH GmbH, Haan, Germany). A 30 mg sample powder was weighed, and 1500 μL of 70% methanol-water internal standard extraction solution (pre-cooled to −20 °C) was added. The mixture was vortexed once every 30 min, with each vortex lasting 30 s, totaling 6 vortexes. After centrifugation for 3 min, the supernatant was aspirated, filtered through a microporous membrane (0.22 μm), and stored in sample vials for UPLC-MS/MS analysis [22].

#### 2.7.2. Liquid Chromatography Conditions

Chromatographic conditions: (1) T3 chromatographic column (Waters Corporation, MA, USA) with (1.8 µm, 2.1 mm × 100 mm); (2) Mobile phase A: ultrapure water (containing 0.1% formic acid); Mobile phase B: acetonitrile (containing 0.1% formic acid); (3) Instrument column temperature: 40 °C; Flow rate: 0.40 mL/min; Injection volume: 4 µL.

#### 2.7.3. Preprocessing of Raw Data

Raw mass spectrometry data were converted to mzML format using Proteo Wizard, and peak extraction, alignment, and retention time correction were performed using the XCMS program (Scripps Research Institute, CA, USA). Peaks with a missing rate > 50% in each group of samples were filtered out. Blank values were filled using a combination of KNN imputation and 1/5 minimum value (for blank values > 50%, 1/5 minimum value was used; for blank values < 50%, KNN imputation was applied). Peak areas were corrected using the Support Vector Regression (SVR) method. The corrected and filtered peaks were subjected to metabolite identification by searching against a laboratory-built database, integrated public databases, prediction databases, and via the metDNA method. Finally, substances with a comprehensive identification score > 0.5 and a coefficient of variation (CV) < 0.5 in quality control (QC) samples were extracted. These substances were then merged in positive and negative modes (retaining those with the highest qualitative grade and the smallest CV value), resulting in the “all_sample_data.xlsx” file. False Discovery Rate (FDR) is obtained by adjusting the *p*-values from significance tests. During the differential expression analysis, the recognized Benjamini–Hochberg adjustment method was applied to correct the original *p*-values from hypothesis testing, with an FDR < 0.05 set as the default threshold. For two-group comparisons, differential metabolites were determined by Variable Importance in the Projection (VIP) (VIP > 1) and absolute Log_2_FC (|Log_2_FC| ≥ 1.0). VIP values were extracted from the Orthogonal Partial Least Squares Discriminant Analysis (OPLS-DA) result, which also contains score plots and permutation plots, and was generated using R package(Auckland University, Auckland, New Zealand) MetaboAnalystR. The data was log-transform (Log_2_) and mean centering before OPLS-DA. A permutation test (200 permutations) was performed to avoid overfitting. Annotation of the identified metabolites was performed using the Kyoto Encyclopedia of Genes and Genomes (KEGG) Compound database (http://www.kegg.jp/kegg/compound/, accessed on 15 August 2025). Annotated metabolites were then mapped to the KEGG Pathway database (http://www.kegg.jp/kegg/pathway.html, accessed on 15 August 2025).

### 2.8. Data Analysis

Excel 2019 software (Microsoft Corporation, Redmond, Washington, DC, USA) was used for data processing, among which the Two-way ANOVA with Tukey was applied for significance analysis. GraphPad Prism software (GraphPad Software, CA, USA) and Adobe Illustrator CC software (Adobe Systems Incorporated, CA, USA) were used to produce various types of graphs.

## 3. Results

### 3.1. Screening of Cultivar for Field Resistance to BSD in Sweet Cherry

**Figure 1:**
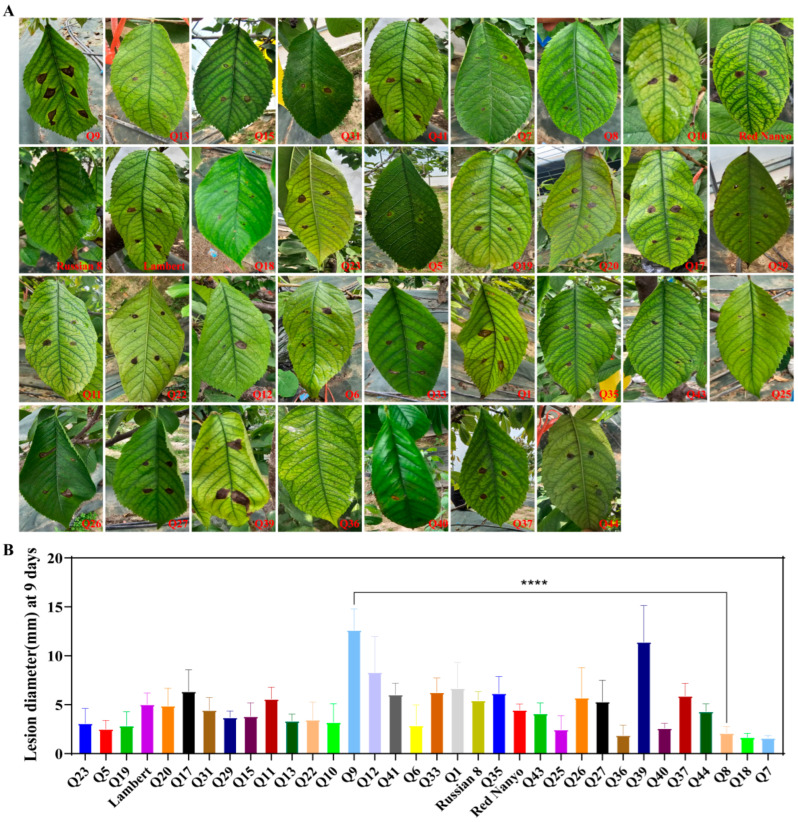
Field resistance phenotypes and statistics of 34 sweet cherry cultivars to *A. alternata*. (**A**) Field phenotypes of sweet cherry leaves at 9 dpi. (**B**) Statistics of the field lesion diameter of sweet cherry leaves at 9 dpi. Note: **** indicates significant difference at the *p* < 0.0001 level.

Field-grown leaves from 34 sweet cherry cultivars (Table S1) exhibited significant differences in resistance to *A. alternata* (Figure 1A), with lesion diameter ranging from 1.61 mm to 12.65 mm at 9 dpi (Figure 1B). With the prolongation of infection days, the lesion diameter increased gradually among the cultivars (Figure S1). At 9 days after inoculation, 9 cultivars displayed high resistance; these cultivars were Q5, Q6, Q7, Q8, Q18, Q19, Q25, Q36, and Q40 (lesion diameter on average ranged from 1.61 mm to 2.89 mm), accounting for 26.47% of the tested cultivars. Only one cultivar Q9 was highly susceptible, representing 2.94% of the tested cultivars.

### 3.2. Confirmation of BSD Resistance in Resistant Sweet Cherry Cultivar Q8 and Susceptible Cultivar Q9

**Figure 2:**
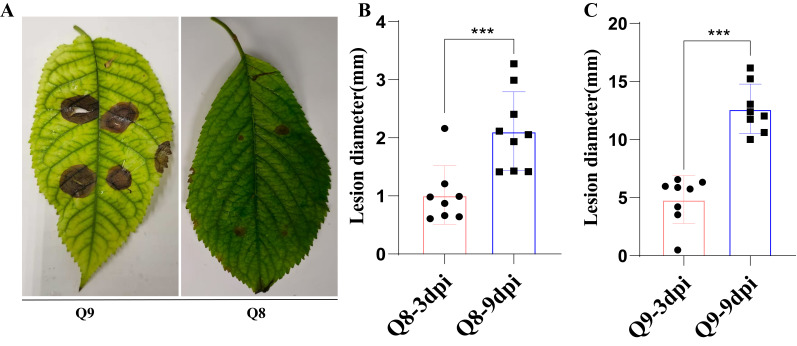
Resistance phenotypes of disease-resistant sweet cherry cultivar Q8 and susceptible Q9 to *A. alternata* at 9 dpi. (**A**) Field phenotypes of sweet cherry cultivars Q8 and Q9 at 9 dpi. (**B**) Field lesion diameter of sweet cherry cultivar Q8 at 3 dpi and 9 dpi. (**C**) Field lesion diameter of sweet cherry cultivar Q9 at 3 dpi and 9 dpi. Note: *** indicate significant difference at the *p* < 0.001 level.

With the prolongation of infection time, the lesion diameter of cultivar Q9 showed a significant increasing trend, while Q8 only exhibited slight lesions (Figure 2A). At the early stage of infection (3 dpi), the average lesion diameter of cultivar Q8 was less than 1 mm (Figure 2B), whereas that of Q9 increased to 4.21 mm (Figure 2C). At the late stage of infection (9 dpi), the average lesion diameter of cultivar Q9 increased notably, reaching 12.65 mm (Figure 2C), while Q8 showed only a slight increase in lesion diameter (Figure 2B). The lesion diameter of sweet cherry cultivar Q8 ranged from 1.87 to 2.25 mm during 11 dpi, while that of cultivar Q9 varied from 7.49 to 18.84 mm (Table S2).

### 3.3. Physiological Changes in Leaves of Resistant Sweet Cherry Cultivar Q8 and Susceptible Cultivar Q9 Under BSD Infection

**Figure 3:**
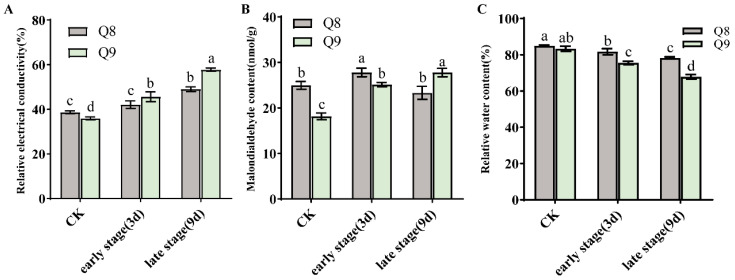
Effects of *A. alternata* infection on the physiological indices of cultivar Q8 and Q9 at 0, 3, and 9 dpi. (**A**) REC values of leaves from sweet cherry cultivars Q8 and Q9 at different infection stages. (**B**) MDA values of leaves from sweet cherry cultivars Q8 and Q9 at different infection stages. (**C**) RWC values of leaves from sweet cherry cultivars Q8 and Q9 at different infection stages. Note: different lowercase letters indicate significant differences at the *p* < 0.05 level.

The leaf REC of both cultivars Q8 and Q9 exhibited a significant (*p* < 0.05) increasing trend (Figure 3A). After leaf infection by BSD, the degree of REC change in cultivar Q9 was greater than that in Q8. As REC serves as an indicator for measuring plant cell membrane permeability, our results indicated that cultivar Q9 undergoes more severe cell damage than Q8. MDA content of cultivar Q9 exhibited a marked (*p* < 0.05) rising trend after BSD infection. In contrast, the MDA content of cultivar Q8 showed minimal fluctuations during the BSD infection: it increased significantly (*p* < 0.05) at the early stage of infection, but returned to the contents of healthy leaves by 9 dpi (Figure 3B). RWC in the leaves of both cultivars Q8 and Q9 displayed a notable (*p* < 0.05) decreasing trend; however, Q8 had a smaller magnitude of RWC change (Figure 3C).

### 3.4. Principal Component Analysis of Metabolites in Sweet Cherry Leaves at Different Infection Stages Under Negative and Positive Modes

**Figure 4:**
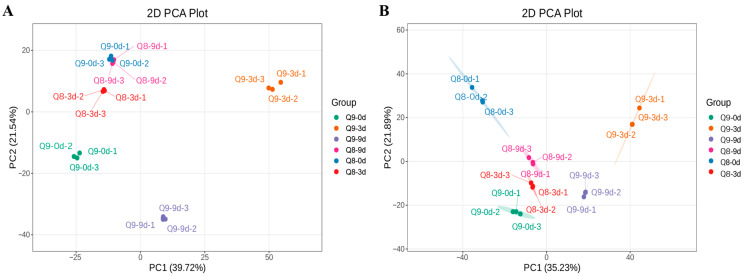
Metabolomic PCA of the effects of *A. alternata* infection on cultivar Q8 and Q9 in negative and positive ion modes. (**A**) PCA score plot under negative ion mode. (**B**) PCA score plot under positive ion mode.

Metabolomics results demonstrated high overlap of the Total Ion Current (TIC) curves in the negative and positive ion modes, with consistent retention times and peak intensities (Figure S2). This indicated robust signal stability of the mass spectrometry system during repeated analyses of the same sample at different time points. A total of 3436 metabolites were detected, including 1738 in the negative ion mode and 1698 in the positive ion mode. (Figure S3). Principal component analysis (PCA) results conducted on 18 samples showed that the first principal component (PC1) accounted for 39.72% of the variance, while the second principal component (PC2) accounted for 21.54%, with a cumulative variance explained of 61.26% for metabolic variance in the negative ion mode. With the exception of the overlap between Q9-0d and Q8-9d samples, the remaining data exhibited significant separation in group-specific metabolite differences, and the intra-group variation among the 3 biological replicates was minimal. (Figure 4A). In the positive ion mode, PC1 and PC2 accounted for 35.23% and 21.89% of the variance, respectively, with a cumulative variance explained of 57.12% (Figure 4B). Samples within each group were clustered together, exhibiting low intra-group variation and significant inter-group separation, indicating that the experiment had high reproducibility.

### 3.5. Statistics of Metabolites in Sweet Cherry Leaves at Different Infection Stages in Negative and Positive Ion Modes

**Figure 5:**
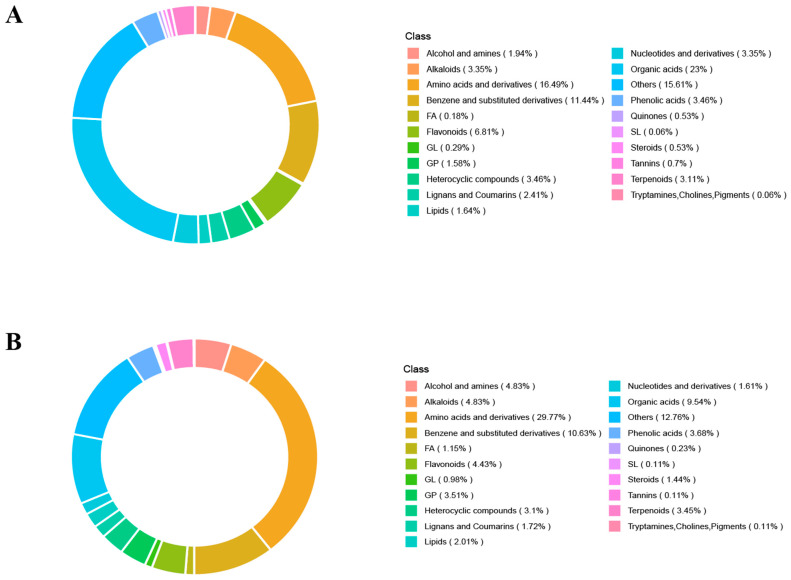
Classification of total metabolites in negative and positive ion modes in the metabolomics of the effects of *A. alternata* infection on cultivar Q8 and Q9. (**A**) Circular chart of metabolite class composition under negative ion mode. (**B**) Circular chart of metabolite class composition under positive ion mode.

In the negative ion mode, organic acids (23%) accounted for the highest proportion of metabolites, followed by amino acids and their derivatives (16.49%), the “Others” category (15.61%), and benzenes and substituted derivatives (11.44%); the total proportion of these four categories reached 66.54% (Figure 5A). In the positive ion mode, amino acids and their derivatives (29.77%) accounted for the largest proportion, followed by “Others” (12.76%), benzenes and substituted derivatives (10.63%), and organic acids(9.54%), with the four categories totaling 62.7% (Figure 5B).

### 3.6. OPLS-DA Analysis of Metabolites in Sweet Cherry Leaves at Different Infection Stages

**Figure 6:**
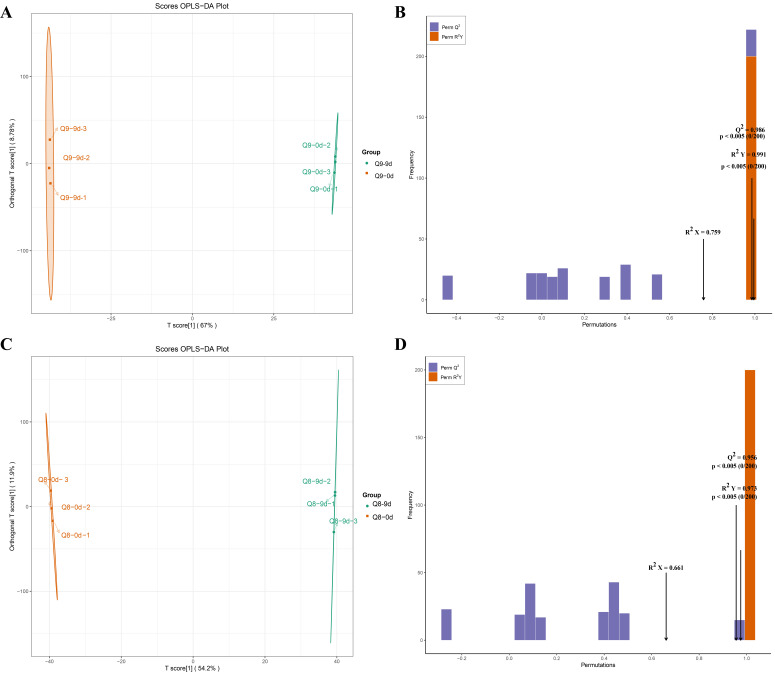
OPLS-DA score plots for the metabolomics of the effects of *A. alternata* infection on cultivar Q9 and Q8 at 0 dpi and 9 dpi. (**A**) OPLS-DA plot of the metabolome of sweet cherry cultivar Q9 at 0 dpi and 9 dpi. (**B**) OPLS-DA permutation plot of the metabolome of sweet cherry cultivar Q9 at 0 dpi and 9 dpi. (**C**) OPLS-DA plot of the metabolome of sweet cherry cultivar Q8 at 0 dpi and 9 dpi. (**D**) OPLS-DA permutation plot of the metabolome of sweet cherry cultivar Q8 at 0 dpi and 9 dpi.

For cultivar Q9, comparison of samples at 0 dpi and 9 dpi revealed significant separation in metabolite profiles between the two groups, indicating substantial inter-group metabolic differences (Figure 6A). The model parameters were R^2^X = 0.759, R^2^Y = 0.991, and Q^2^ = 0.986, indicating that the model explained 75.9% of the total variance and 99.1% of the inter-group differences, with a predictive ability of 98.6% (Figure 6B). For cultivar Q8, comparison of samples at 0 dpi and 9 dpi demonstrated obvious separation in the metabolite profiles between the groups (Figure 6C). The model parameters were R^2^X = 0.661, R^2^Y = 0.973, and Q^2^ = 0.956, indicating that the model explained 66.1% of the total variance and 97.3% of the inter-group differences, with a predictive ability of95.6% (Figure 6D). This verifies that the OPLS-DA model constructed in this study performed well and could effectively elucidate the metabolic differences between the two sample groups.

### 3.7. Volcano Plot and Heatmap Analysis of Metabolites in Sweet Cherry Leaves at Different Infection Stages

**Figure 7:**
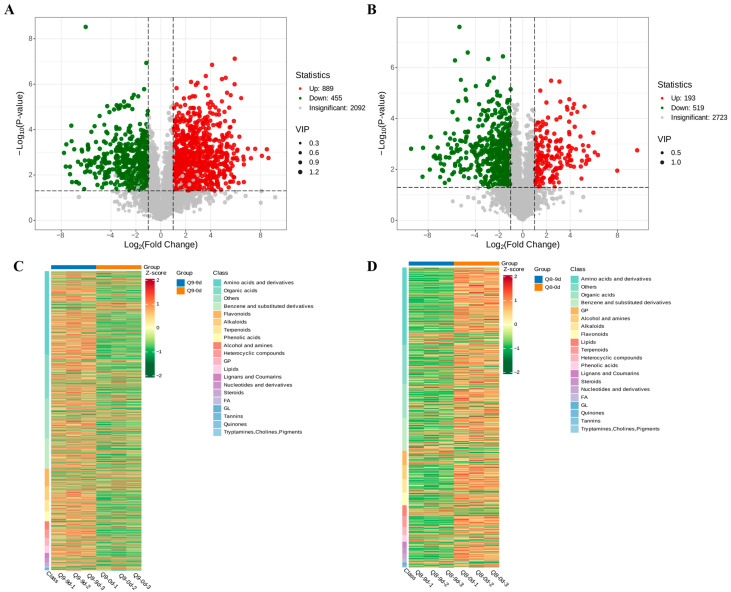
Volcano plots and classification heatmaps for the metabolomics of the effects of *A. alternata* infection on cultivar Q9 and Q8 at 0 dpi and 9 dpi. (**A**) Volcano plot of DAMs of sweet cherry cultivar Q9 at 0 dpi and 9 dpi. (**B**) Volcano plot of DAMs of sweet cherry cultivar Q8 at 0 dpi and 9 dpi. (**C**) Heatmap of DAMs of sweet cherry cultivar Q9 at 0 dpi and 9 dpi. (**D**) Heatmap of DAMs of sweet cherry cultivar Q8 at 0 dpi and 9 dpi.

For cultivar Q9 leaves, comparative analysis of 0 dpi and 9 dpi samples identified 889 significantly up-regulated and 455 significantly down-regulated metabolites (*p* < 0.05, |log_2_ FC| ≥ 1). The primary categories of these differentially accumulated metabolites (DAMs) were amino acids and their derivatives, as well as organic acids (Figure 7A,C). For cultivar Q8 leaves, comparative analysis of 0 dpi and 9 dpi samples identified 193 notably up-regulated and 519 significantly down-regulated metabolites (*p* < 0.05, |log_2_ FC| ≥ 1). The primary categories of these DAMs were amino acids and their derivatives, along with the “Others” category (Figure 7B,D).

### 3.8. Identification of Active Metabolites Involved in BSD Resistance and Their Pathway Analysis in Q8

**Figure 8:**
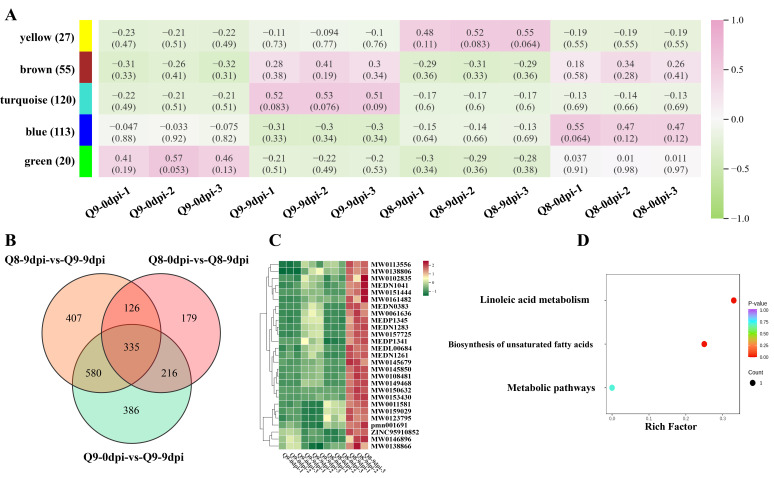
Mining of differential metabolites and KEGG pathway enrichment analysis of the effects of *A. alternata* infection on cultivar Q8 and Q9. (**A**) WGCNA plot of key DAMs in sweet cherry cultivars Q8 and Q9 at 0 dpi and 9 dpi. (**B**) Venn plot of key DAMs in sweet cherry cultivars Q8 and Q9 under infection. (**C**) Clustered heatmap of key DAMs in sweet cherry cultivars Q8 and Q9 at 0 dpi and 9 dpi. (**D**) KEGG of key DAMs in sweet cherry cultivars Q8 and Q9.

Venn analysis of three contrasts (Q8 0 dpi vs. 9 dpi, Q9 0 dpi vs. 9 dpi, Q8 vs. Q9 at 9 dpi) yielded 335 shared DAMs (Figure 8B). Subsequently, Weighted Gene Co-expression Network Analysis (WGCNA) was conducted on these shared DAMs, which identified 27 DAMs up-regulated in the disease-resistant cultivar Q8 at 9 dpi (Figure 8A). Heatmap analysis of these up-regulated DAMs showed that they were mainly categorized into two groups: (1) benzenes and substituted derivatives; (2) organic acids and their lipids (Figure 8C). Furthermore, KEGG pathway analysis was conducted on these up-regulated DAMs, which revealed that they were mainly enriched in two pathways: linoleic acid metabolism and unsaturated fatty acid metabolism (Figure 8D).

### 3.9. Metabolic Mechanisms Underlying BSD Resistance in Q8 and Susceptibility in Q9

**Figure 9:**
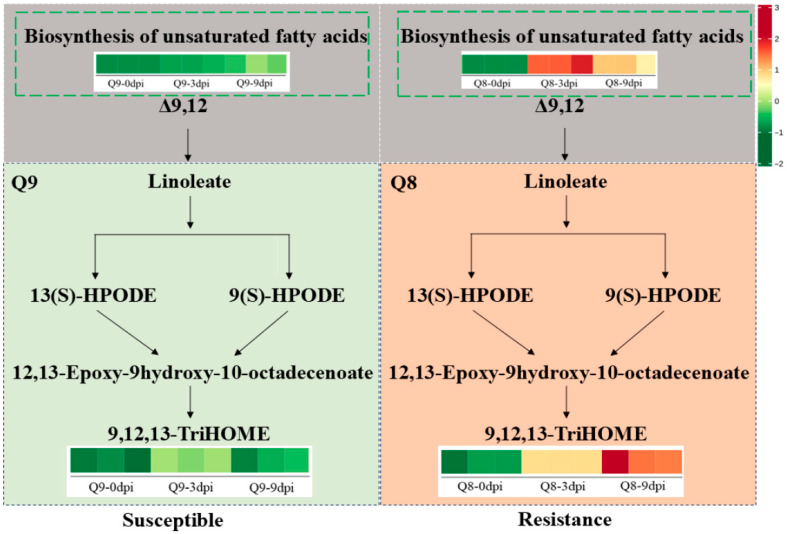
Comparative metabolic mechanism diagram of disease-resistant cultivar Q8 and susceptible Q9 in response to *A. alternata* infection.

A pathway diagram was constructed to elucidate the mechanism of BSD resistance in sweet cherry cultivar Q8 (Figure 9). In the disease-susceptible cultivar Q9, following infection by BSD, the unsaturated fatty acid metabolism pathway exhibited low activity, which resulted in a low content of Δ9,12 (Linoleic acid). A low Δ9,12 content further induced a reduction in the downstream pathway of linoleic acid metabolism, failing to produce sufficient Pinellic acid (a potential disease-resistant active metabolite) and possibly making Q9 susceptible to BSD. Conversely, upon BSD infection, the disease-resistant cultivar Q8 exhibited high activity in the unsaturated fatty acid metabolism pathway, thus accumulating high levels of Δ9,12. Elevated Δ9,12 content in cultivar Q8 promoted higher downstream metabolite levels in linoleic acid metabolism, which further boosted the synthesis of the potential disease-resistant active metabolite Pinellic acid. Thus, the significant accumulation of Pinellic acid in cultivar Q8 suggests that it may confer resistance to BSD.

## 4. Discussion

In this study, we screened the differences in resistance to BSD among sweet cherry cultivars in Qinghai Province, China, and found that the resistance of sweet cherry exhibited temporal complexity and significant cultivar-specific differences. Sweet cherry has been reported to show notable cultivar differences in resistance to bacterial diseases, which implies that it may also have distinct cultivar-specific resistance to other pathogens [23].

This study quantified three key physiological indicators (REC, MDA, and RWC) at 0, 3, and 9 dpi to investigate the responses of resistant cultivar Q8 and susceptible cultivar Q9 to pathogen infection, and notable differences were identified between the two cultivars. For REC, cultivar Q8 exhibited a slower increase rate and maintained stable membrane integrity during the first 3 dpi, while Q9 showed sustained REC elevation, reflecting progressive membrane disruption. This trend is consistent with rice blast studies reporting greater membrane damage in susceptible genotypes [24]. For cultivar Q8, its MDA content exhibited an initial increase followed by a decline, a trend that implies the activation of the antioxidant system to alleviate lipid peroxidation-induced damage [25]. In contrast, cultivar Q9 displayed continuous MDA accumulation, indicating exacerbated oxidative damage, which is consistent with findings in clubroot-resistant rapeseed [26]. Additionally, the RWC of Q8 decreased to a smaller extent than that of Q9 at all infection stages. These results indicate that after *A. alternata* infection, the physiological status of the resistant cultivar Q8 was less affected and could recover more rapidly compared with Q9. Previous studies have demonstrated that when the resistant cherry cultivar RC and the susceptible cultivar SC were infected by *A. alternata*, the MDA content of RC increases to a smaller extent and recovers in the later stage, while that of SC continues to rise [27,28]. Research on other plant species has also shown that after pathogen infection, resistant cultivars exhibit smaller changes in electrical conductivity and RWC [29]. These findings are consistent with the results of our study.

Following pathogen inoculation, resistant cultivars typically display minor lesions or no observable lesions, while susceptible cultivars develop large-scale lesions. This phenomenon occurs because the effectors secreted by pathogens are recognized by plant resistance proteins (R proteins), thereby conferring enhanced disease resistance to the host plants [30,31,32,33]. In this study, we observed a smaller lesion area (lesion diameter ≤ 2 mm) on sweet cherry cultivar Q8 at 9 dpi, suggesting that Q8 may contain resistance capable of recognizing the effectors of *A. alternata*. In subsequent studies, using these effectors as baits to clone resistance genes from cultivar Q8 will lay a foundation for disease-resistant breeding of sweet cherry.

Metabolomics is a crucial approach for identifying key defence-related metabolites and their regulatory genes [5]. Previous studies have confirmed that metabolic pathways such as linolenic acid metabolism and unsaturated fatty acid metabolism are involved in plant disease resistance [34,35,36,37]. For instance, Visha Rathod‘s study found that when peanuts were infected by *Puccinia arachidis*, linolenic acid metabolism was highly enriched in resistant peanut cultivars compared with susceptible ones [38]. Similarly, another study reported that the resistant sweet cherry cultivar RC promotes the jasmonic acid (JA) signaling-mediated disease resistance process through linolenic acid metabolism when infected by *A. alternata* [27]. Consistent with these previous findings, our study also revealed that the linolenic acid metabolism and unsaturated fatty acid biosynthesis pathways are highly active in the resistant cultivar Q8 after *A. alternata* infection. It has been reported that members of the TriHOME family are involved in inflammatory responses in animals [39]. Specifically, Pinellic acid plays a critical role in the defense response of rice against *Magnaporthe oryzae* [40]. As an oxylipin-derived signaling molecule, Pinellic acid plays a pivotal role in rice immunity, being rapidly synthesized upon recognition of the fungal pathogen *Magnaporthe oryzae* [41]. It serves as a critical component of the rice defense system against blast disease via a multifaceted mechanism, which includes direct antifungal activity against the pathogen, modulation of localized programmed cell death to restrict hyphal propagation, and activation of a systemic defense gene expression network [42]. We observed significant accumulation of Pinellic acid in Q8 during infection. Therefore, we hypothesize that this metabolite is likely involved in the disease resistance of sweet cherry against *A. alternata*. Future work should validate the defensive role of Pinellic acid and identify the genes regulating its biosynthesis.

## 5. Conclusions

Sweet cherry is significantly threatened by BSD in its cultivation, which leads to yield loss and compromised fruit quality. This study employed field-based disease resistance screening, physiological assays, and metabolomic analyses to elucidate the metabolic foundation of disease resistance in sweet cherry. This study successfully identified 9 highly resistant cultivars and one highly susceptible cultivar from 34 sweet cherry resources. Physiological assays revealed significant differences in MDA content, RWC, and REC between resistant and susceptible sweet cherry cultivars. Our metabolome data reveal, for the first time, that linolenic-acid and unsaturated-fatty-acid metabolism are integral to sweet-cherry defense against BSD. Notably, Pinellic acid was found to be significantly enriched in the resistant cultivar Q8, indicating that it may act as a bioactive signaling molecule or antimicrobial agent directly involved in mediating plant immune responses. The present study not only delineates part of the metabolic basis underlying BSD resistance in sweet cherry but also lays a robust theoretical and material foundation for breeding BSD-resistant sweet cherry cultivars via molecular marker-assisted selection (MAS) and metabolic engineering approaches.

## Data Availability

The original contributions presented in this study are included in the article/supplementary material. Further inquiries can be directed to the corresponding authors.

## Supplementary Materials

The following supporting information can be downloaded at: https://www.mdpi.com/article/10.3390/biom15121730/s1, Figure S1. Direct statistics of lesions on 34 sweet cherry cultivars infected by *A. alternata* at 5, 7, and 11 dpi in the field. Figure S2. Metabolomic quality control in negative and positive ion modes for the effects of *A. alternata* infection on cultivar Q8 and Q9. Figure S3. Metabolomic statistics of total metabolites in negative and positive ion modes. Table S1. Names and origin details of 34 sweet cherry cultivars. Table S2. Statistics of lesion diameter of 34 sweet cherry cultivars at 5, 7, 9, 11 dpi.

## Abbreviations

The following abbreviations are used in this manuscript:

dpi	days post-infection
BSD	Brown spot disease
REC	Relative Electrical Conductivity
MDA	Malondialdehyde
RWC	Relative Water Content
PCA	Principal component analysis
SVR	Support Vector Regression
FDR	False Discovery Rate
VIP	Variable Importance in the Projection
OPLS-DA	Orthogonal Partial Least Squares Discriminant Analysis
PDA	Potato Dextrose Agar
DAMs	Differentially Accumulated Metabolites
JA	Jasmonic acid
KEGG	Kyoto Encyclopedia of Genes and Genomes
WGCNA	Weighted Gene Co-expression Network Analysis
MAS	Marker-assisted selection

## References

[1] Wang Y.Y., Xiao Y.Q., Sun Y., Zhang X., Du B.Y., Turupu M., Yao Q.S., Gai S.L., Tong S., Huang J. (2023). Two B-box proteins, *PavBBX6/9*, positively regulate light-induced anthocyanin accumulation in sweet cherry. Plant Physiol..

[2] Zhai Z.F., Xiao Y.Q., Wang Y.Y., Sun Y.T., Peng X., Feng C., Zhang X., Du B.Y., Zhou X., Wang C. (2022). Abscisic acid-responsive transcription factorsPavDof2/6/15 mediate fruit softening in sweet cherry. Plant Physiol..

[3] Shastry A., Dunham-Snary K. (2023). Metabolomics and mitochondrial dysfunction in cardiometabolic disease. Life Sci..

[4] Xiao P., Qu J., Wang Y., Fang T., Xiao W., Wang Y.L., Zhang Y., Khan M., Chen Q., Xu X.Y. (2024). Transcriptome and metabolome atlas reveals contributions of sphingosine and chlorogenic acid to cold tolerance in *Citrus*. Plant Physiol..

[5] Tian M.J., Sun Y.R., Zhang G.D., Xu Y.F., Zhu J., Huang W.W., Wang Y.Z., Zhang B.C., Li Z.Y., Lin S.Y. (2025). Metabolomics navigates natural variation in pathogen-induced secondary metabolism across soybean cultivar populations. Proc. Natl. Acad. Sci. USA.

[6] Liu L.D., Sun Y., Wen C.X., Jiang T., Tian W., Xie X.L., Cui X.S., Lu R.K., Feng J.X., Jin A.H. (2022). Metabolome analysis of genus *Forsythia* related constituents in *Forsythia suspensa* leaves and fruits using UPLC-ESI-QQQ-MS/MS technique. PLoS ONE.

[7] Qi X.L., Liu L.F., Liu C.L., Song L.L., Dong Y.X., Chen L., Li M. (2023). Sweet cherry AP2/ERF transcription factor, *PavRAV2*, negatively modulates fruit size by directly repressing *PavKLUH* expression. Physiol. Plant..

[8] Qi X.L., Liu C.L., Song L.L., Dong Y.X., Chen L., Li M. (2022). A sweet cherry glutathione s-transferase gene, *PavGST1*, plays a central role in fruit skin coloration. Cells.

[9] Hou Q.D., Shen T.J., Yu R.R., Deng H., Wen X.P., Qiao G. (2023). Functional analysis of sweet cherry *PavbHLH106* in the regulation of cold stress. Plant Cell Rep..

[10] Ricardo-Rodrigues S., Laranjo M., Agulheiro-Santos A.C. (2023). Methods for quality evaluation of sweet cherry. J. Sci. Food Agric..

[11] Wu F.L., Qu D.H., Zhang X., Sun Y., Wang J.T., Zhu D.Z., Yang L.N., Liu X., Tian W., Wang L. (2023). *PaLectinL7* enhances salt tolerance of sweet cherry by regulating lignin deposition in connection with PaCAD1. Tree Physiol..

[12] Qi X.L., Dong Y.X., Liu C.L., Song L.L., Chen L., Li M. (2022). The *PavNAC56* transcription factor positively regulates fruit ripening and softening in sweet cherry (*Prunus avium*). Physiol. Plant.

[13] Vega E.N., García-Herrera P., Ciudad-Mulero M., Dias M.I., Matallana-González M.C., Cámara M., Tardío J., Molina M., Pinela J., Pires T.C.S.P. (2023). Wild sweet cherry, strawberry and bilberry as underestimated sources of natural colorants and bioactive compounds with functional properties. Food Chem..

[14] Orfanidou C.G., Katsiani A., Candresse T., Marais A., Gkremotsi T., Drogoudi P., Kazantzis K., Katis N.I., Maliogka V.I. (2023). Identification of divergent isolates of cherry latent virus 1 in Greek sweet cherry orchards. Arch. Virol..

[15] Probst C., Pandey B., Swamy P., Grove G. (2021). Factors affecting the infection of sweet cherry (*Prunus avium*) fruit by *Podosphaera cerasi*. Plant Dis..

[16] Nejad M.S., Najafabadi N.S., Aghighi S., Zargar M., Bayat M., Pakina E. (2024). Green synthesis of silver nanoparticles by sweet cherry and its application against cherry spot disease. Heliyon.

[17] Wang J.W., Zhu D.Z., Liu Q.Z., Davis R.E., Zhao Y. (2014). First report of sweet cherry virescence disease in China and its association with infection by a ‘Candidatus *Phytoplasma ziziphi*’-related strain. Plant Dis..

[18] Liu Q., Ning N.N., Ma Y.Q., Guo Q.Y. (2020). Isolation and identification of the pathogen causing cherry leaf spot in Qinghai province. Plant Prot..

[19] Arndt S.K., Irawan A., Sanders G.J. (2015). Apoplastic water fraction and rehydration techniques introduce significant errors in measurements of relative water content and osmotic potential in plant leaves. Physiol. Plant..

[20] Shan G.M., Li X.T., Liu P., Zhou J.Y., Wang Y.S., Gao R. (2025). Morphological characteristics and cold tolerance analysis of ground cover chrysanthemums Yannong Guiyou’ × ‘Yannong Maohua’ and their hybrids. North. Hortic..

[21] Yuan H.R., Cheng M.X., Wang R.H., Wang Z.K., Fan F.F., Wang W., Si F.F., Gao F., Li S.Q. (2024). *miR396b/GRF6* module contributes to salt tolerance in rice. Plant Biotechnol. J..

[22] Chen Y.H., Zhang R.P., Song Y.M., He J.M., Sun J.H., Bai J.F., An Z.L., Dong L.J., Zhan Q.M., Abliz Z. (2009). RRLC-MS/MS-based metabonomics combined with in-depth analysis of metabolic correlation network: Finding potential biomarkers for breast cancer. Analyst.

[23] Hulin M.T., Vadillo D.A., Cossu F., Lynn S., Russell K., Neale H.C., Jackson R.W., Arnold D.L., Mansfield J.W., Harrison R.J. (2022). Identifying resistance in wild and ornamental cherry towards bacterial canker caused by *Pseudomonas syringae*. Plant Pathol..

[24] Shah N., Li Q., Xu Q., Liu J., Huang F., Zhan Z.X., Qin P., Zhou X.P., Yu W.L., Zhang C.Y. (2020). *CRb* and *PbBa8.1* synergically increases resistant genes expression upon infection of *Plasmodiophora brassicae* in *Brassica napus*. Genes.

[25] Liang D., Yang D.Y., Li T., Zhu Z., Yan B.X., He Y., Li X.Y., Zhai K.R., Liu J.Y., Kawano Y. (2025). A PRA-Rab trafficking machinery modulates NLR immune receptor plasma membrane microdomain anchoring and blast resistance in rice. Sci. Bull..

[26] Li Z.B., Luo W.D., Xie H.T., Mo C.P., Qin B.X., Zhao Y.G., Chen X., Zhang S.B., Zhao Y.L., Wang M.C. (2025). Reovirus infection results in rice rhizosphere microbial community reassembly through metabolite-mediated recruitment and exclusion. Microbiome.

[27] Pan L.Y., Zhou J., Sun Y., Qiao B.X., Wan T., Guo R.Q., Zhang J., Shan D.Q., Cai Y.L. (2023). Comparative transcriptome and metabolome analyses of cherry leaves spot disease caused by *Alternaria alternata*. Front. Plant Sci..

[28] Zhang Y.J., Dong W.K., Zhao C.X., Ma H.L. (2022). Comparative transcriptome analysis of resistant and susceptible Kentucky bluegrass varieties in response to powdery mildew infection. BMC Plant Biol..

[29] Zhang D., Tang J., Wei K., Jia S.G., Jiang Y.W., Cai H.W., Mao P.S., Li M.L. (2022). Physiological and molecular responses of *Zoysia japonica* to rust infection. Int. J. Mol. Sci..

[30] Sun Y., Wang Y., Zhang X.X., Chen Z.D., Xia Y.Q., Wang L., Sun Y.J., Zhang M.M., Xiao Y., Han Z.F. (2022). Plant receptor-like protein activation by a microbial glycoside hydrolase. Nature.

[31] Ma Z.C., Song T.Q., Zhu L., Ye W.W., Wang Y., Shao Y.Y., Dong S.M., Zhang Z.G., Dou D.L., Zheng X.B. (2015). A *Phytophthora sojae* glycoside hydrolase 12 protein is a major virulence factor during soybean infection and is recognized as a PAMP. Plant Cell.

[32] Prautsch J., Erickson J.L., Özyürek S., Gormanns R., Franke L., Lu Y., Marx J., Niemeyer F., Parker J.E., Stuttmann J. (2023). Effector XopQ-induced stromule formation in *Nicotiana benthamiana* depends on ETI signaling components ADR1 and NRG1. Plant Physiol..

[33] Gu K.Y., Yang B., Tian D.S., Wu L.F., Wang D.J., Sreekala C., Yang F., Chu Z.Q., Wang G.L., White F.F. (2005). R gene expression induced by a type-III effector triggers disease resistance in rice. Nature.

[34] Nie X.H., Zhao S.Q., Hao Y.Q., Gu S., Zhang Y., Qi B.X., Xing Y., Qin L. (2023). Transcriptome analysis reveals key genes involved in the resistance to *Cryphonectria parasitica* during early disease development in Chinese chestnut. BMC Plant Biol..

[35] Li Y.H., Qiu L.N., Liu X.Y., Zhang Q., Zhuansun X.X., Fahima T., Krugman T., Sun Q.X., Xie C.J. (2020). Glycerol-induced powdery mildew resistance in wheat by regulating plant fatty acid Metabolism, plant hormones cross-talk, and pathogenesis-related genes. Int. J. Mol. Sci..

[36] Li M.R., Qi X., Li D., Wu Z.Q., Liu M.Y., Yang W.G., Zang Z.Y., Jiang L.Y. (2024). Comparative transcriptome analysis highlights resistance regulatory networks of maize in response to *Exserohilum turcicum* infection at the early stage. Physiol. Plant..

[37] Zhu Y.T., Hu X.Q., Wang P., Gao L.Y., Pei Y.K., Ge Z.Y., Ge X.Y., Li F.G., Hou Y.X. (2021). *GhPLP2* positively regulates cotton resistance to *Verticillium Wilt* by modulating fatty acid accumulation and jasmonic acid signaling pathway. Front. Plant Sci..

[38] Rathod V., Rathod K., Tomar R.S., Tatamiya R., Hamid R., Jacob F., Munshi N.S. (2023). Metabolic profiles of peanut (*Arachis hypogaea* L.) in response to *Puccinia arachidis* fungal infection. BMC Genom..

[39] Fuchs D., Tang X., Johnsson A.K., Dahlén S.E., Hamberg M., Wheelock C.E. (2020). Eosinophils synthesize trihydroxyoctadecenoic acids (TriHOMEs) via a 15-lipoxygenase dependent process. Biochim. Biophys. Acta Mol. Cell Biol. Lipids.

[40] Kato T., Hirukawa T., Yano M. (1994). Synthesis of (E)-9,10,13-Trihydroxy-11-octadecenoic Acids. Bull. Chem. Soc. Jpn..

[41] Liu W.D., Liu J.L., Triplett L., Leach J.E., Wang G.L. (2014). Novel insights into rice innate immunity against bacterial and fungal pathogens. Annu. Rev. Phytopathol..

[42] Miura A., Kuwahara S. (2009). A concise synthesis of pinellic acid using a cross-metathesis approach. Tetrahedron.

