# Supplementary material for: Metabolomics Reveals Resistance-Related Secondary Metabolism in Sweet Cherry Infected by Alternaria alternata
Source: Biomolecules. 2025 Dec 12;15(12):1730. doi: 10.3390/biom15121730 (PMC12730904; doi:10.3390/biom15121730)
Supplement: Supplementary file 1 [file biomolecules-15-01730-s001.zip › biomolecules-3969177-supplementary.pdf]

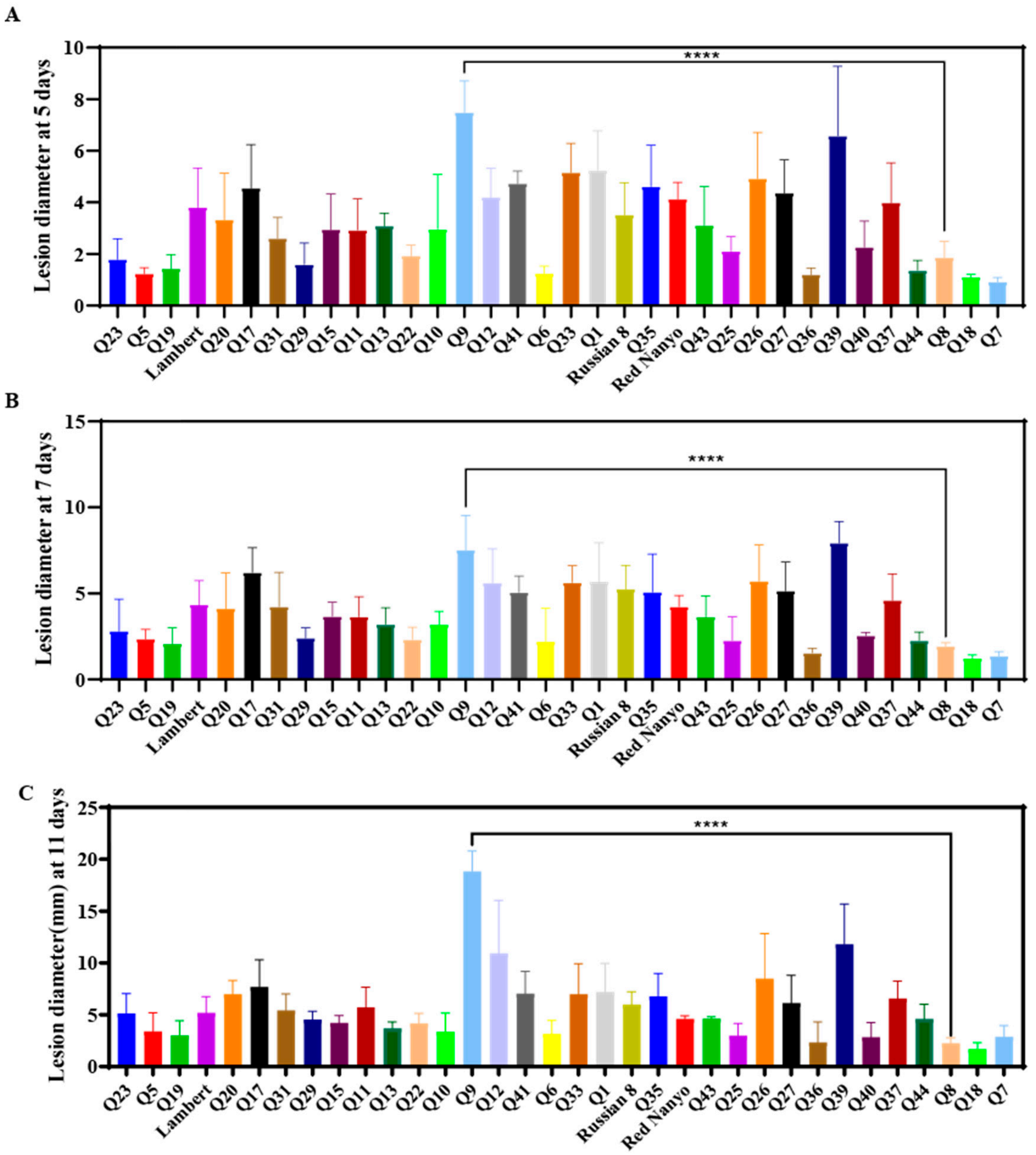

Figure S1 Direct statistics of lesions on 34 sweet cherry cultivars infected by *A. alternata* at 5, 7, and 11 dpi in the field. (**A**) Statistics of field lesion diameter of sweet cherry leaves at 5 dpi. (**B**) Statistics of field lesion diameter of sweet cherry leaves at 7 dpi. (**C**) Statistics of field lesion diameter of sweet cherry leaves at 11 dpi.

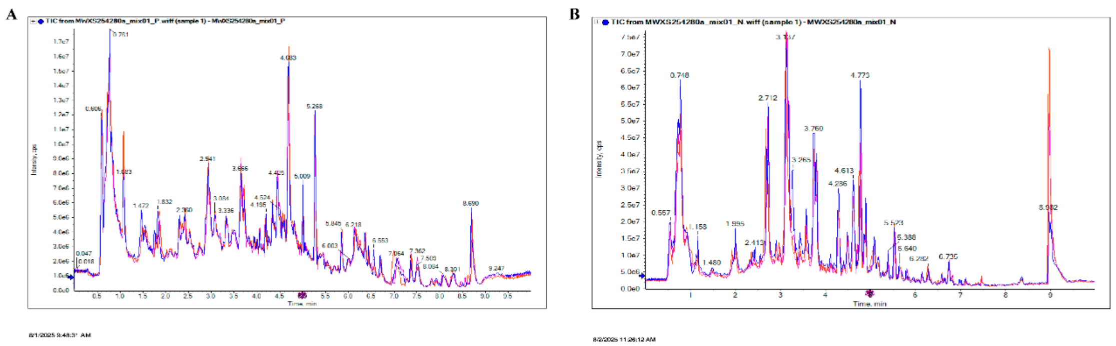

Figure S2 Metabolomic quality control in negative and positive ion modes for the effects of *A. alternata* infection on cultivar Q8 and Q9. (**A**) TIC chromatogram of quality control samples under negative ion mode. (**B**) TIC chromatogram of quality control samples under positive ion mode.

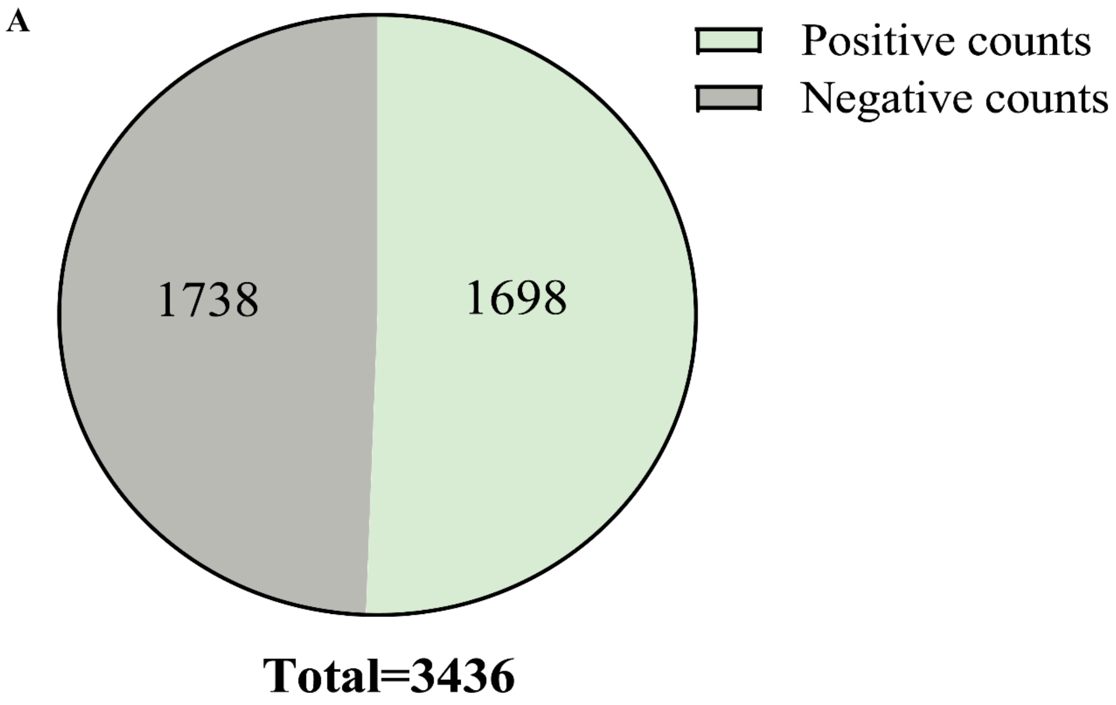

Figure S3 Metabolomic statistics of total metabolites in negative and positive ion modes.

Table S1. Names and origin details of 34 sweet cherry cultivars

Number	Cultivar Name	Origin	Number	Cultivar Name	Origin
Q1	Ambrunes	Spain	Q7	Kristen	USA
Q6	PC8011-4	Qinghai Province, China	Q10	Hedelfingen	UK
Q9	Sparkle	USA	Q13	Qingying 1	Qinghai Province, China
Q12	Ebony	USA	Q17	Corum	USA
Q19	Schnider	Germany	Q20	PC8011-5	Qinghai Province, China
Q22	Qingying 3	Qinghai Province, China	Q23	Olympus	USA
Q25	Gil peck	USA	Q26	Napoleon	USA
Q31	Bing	USA	Q29	Sweet Heart	Canada
Q36	Van	Canada	Q33	Summit	Canada
Q39	Summit	Canada	Q37	Sunburst	Canada
Q5	Cashmere	USA	Q40	Emperor Francis	France
Q8	Saylor	USA	Q43	White Gold	USA
Q11	Moreau	France	Q27	Early Robin	USA
Q15	Qingying 2	Qinghai Province, China	Q41	Attika	USA
Q18	Skeena	Canada		Russia 8	Russia
Q35	Utah Giant	USA		Red Nanyo	Japan
Q44	Windsor	USA		Lambert	USA

Table S2. Statistics of lesion diameter of 34 sweet cherry cultivars at 5, 7, 9, 11 dpi

Cultivars	Lesion diameter(mm) at 5 dpi	Lesion diameter(mm) at 7 dpi	Lesion diameter(mm) at 9 dpi	Lesion diameter(mm) at 11 dpi
Q23	1.79±0.8	2.81±1.86	3.1±1.51	5.14±1.91
Q5	1.24±0.23	2.36±0.56	2.53±0.87	3.4±1.8
Q19	1.43±0.54	2.08±0.91	2.85±1.43	3.02±1.41
Lambert	3.82±1.51	4.34±1.41	5.05±1.15	5.17±1.56
Q20	3.33±1.8	4.12±2.07	4.91±1.76	6.98±1.32
Q17	4.55±1.69	6.21±1.44	6.38±2.19	7.67±2.62
Q31	2.6±0.82	4.23±1.99	4.45±1.29	5.42±1.6
Q29	1.6±0.83	2.41±0.6	3.72±0.64	4.53±0.81
Q15	2.95±1.37	3.67±0.83	3.83±1.37	4.22±0.72
Q11	2.93±1.2	3.65±1.16	5.6±1.2	5.72±1.95
Q13	3.09±0.49	3.21±0.95	3.36±0.69	3.7±0.61
Q22	1.93±0.41	2.31±0.72	3.48±1.8	4.16±0.98
Q10	2.96±2.12	3.19±0.76	3.2±1.88	3.37±1.81
Q9	7.49±1.22	7.53±2.01	12.65±2.12	18.84±1.95
Q12	4.2±1.12	5.62±1.97	8.33±3.62	10.92±5.1
Q41	4.73±0.48	5.06±0.94	6.05±1.15	7.03±2.15
Q6	1.26±0.27	2.2±1.93	2.89±2.09	3.16±1.29
Q33	5.15±1.14	5.64±0.98	6.27±1.46	6.96±2.97
Q1	5.24±1.53	5.69±2.25	6.69±2.65	7.19±2.76
Russian 8	3.51±1.25	5.26±1.29	5.44±0.91	5.99±1.23
Q35	4.62±1.6	5.08±2.21	6.18±1.71	6.79±2.2
Red Nanyo	4.13±0.59	4.23±0.65	4.48±0.6	4.62±0.27
Q43	3.11±1.5	3.65±1.22	4.11±1.06	4.64±0.17
Q25	2.11±0.55	2.27±1.36	2.48±1.41	2.97±1.18
Q26	4.92±1.79	5.72±2.1	5.73±3.05	8.49±4.34
Q27	4.36±1.28	5.15±1.68	5.33±2.16	6.12±2.7
Q36	1.19±0.26	1.55±0.28	1.9±1.03	2.33±2
Q39	6.58±2.69	7.93±1.24	11.43±3.71	11.8±3.87
Q40	2.26 ± 0.83	2.54 ± 0.16	2.61 ± 0.41	2.82±1.41
Q37	3.99±1.54	4.59±1.54	5.91±1.28	6.57±1.7
Q44	1.37±0.38	2.27±0.48	4.33±0.76	4.61±1.4
Q8	1.87±0.62	1.93±0.2	2.11±0.64	2.25±0.52
Q18	1.11±0.11	1.23±0.2	1.67±0.41	1.7±0.62
Q7	0.92±0.17	1.36 ± 0.26	1.61±0.24	2.87±1.08
